# Establishment of optimized in vitro disinfection protocol of *Pistacia vera* L. explants mediated a computational approach: multilayer perceptron–multi−objective genetic algorithm

**DOI:** 10.1186/s12870-022-03674-x

**Published:** 2022-07-05

**Authors:** Najet Gammoudi, Kamel Nagaz, Ali Ferchichi

**Affiliations:** 1 Arid and Oases Cropping Laboratory, Arid Lands Institute (IRA), 4119 Medenine, Tunisia; 2 National Institute of Agronomy of Tunis, 43 Charles Nicolle, 1082 Tunis, Tunisia

**Keywords:** In vitro culture, Artificial neural network, Multi−objective genetic algorithm, Optimization, Disinfection, *Pistacia vera*

## Abstract

**Background:**

Contamination−free culture is a prerequisite for the success of in vitro − based plant biotechnology. Aseptic initiation is an extremely strenuous stride, particularly in woody species. Meanwhile, over−sterilization is potentially detrimental to plant tissue. The recent rise of machine learning algorithms in plant tissue culture proposes an advanced interpretive tool for the combinational effect of influential factors for such in vitro − based steps.

**Results:**

A multilayer perceptron (MLP) model of artificial neural network (ANN) was implemented with four inputs, three sterilizing chemicals at various concentrations and the immersion time, and two outputs, disinfection efficiency (DE) and negative disinfection effect (NDE), intending to assess twenty−seven disinfection procedures of *Pistacia vera* L. seeds. Mercury chloride (HgCl_2_; 0.05–0.2%; 5–15 min) appears the most effective with 100% DE, then hydrogen peroxide (H_2_O_2_; 5.25–12.25%; 10–30 min) with 66–100% DE, followed by 27–77% DE for sodium hypochlorite (NaOCl; 0.54–1.26% w/v; 10–30 min). Concurrently, NDE was detected, including chlorosis, hard embryo germination, embryo deformation, and browning tissue, namely, a low repercussion with NaOCl (0–14%), a moderate impact with H_2_O_2_ (6–46%), and pronounced damage with HgCl_2_ (22–100%). Developed ANN showed *R* values of 0.9658, 0.9653, 0.8937, and 0.9454 for training, validation, testing, and all sets, respectively, which revealed the uprightness of the model. Subsequently, the model was linked to multi−objective genetic algorithm (MOGA) which proposed an optimized combination of 0.56% NaOCl, 12.23% H_2_O_2_, and 0.068% HgCl_2_ for 5.022 min. The validation assay reflects the high utility and accuracy of the model with maximum DE (100%) and lower phytotoxicity (7.1%).

**Conclusion:**

In one more case, machine learning algorithms emphasized their ability to resolve commonly encountered problems. The current successful implementation of MLP–MOGA inspires its application for more complicated plant tissue culture processes.

## Background

Plant tissue culture is a well−established strategy for the plant genetic breeding, the production of biologically active compounds, and the conservation and mass propagation of various species [1, 2]. It involves oversensitive, precise, and multi−variables processes considering the extended assortment of in vitro based−plant biotechnologies, for instance, organogenesis, somatic embryogenesis, somatic hybridization, haplodiploidization, hairy root culture, in vitro secondary metabolites production, *Agrobacterium* − mediated gene transformation, and recently as a crucial step in genome editing technologies [3–6]. In vitro cell fate is the outcome of the dynamic interaction mainly of three levels, environmental signal inputs and physical stimuli that act as initial triggers of regeneration, epigenetic and transcriptional cellular responses to those triggers leading to cellular reprogramming, and molecules that manage the development of the new stem cell niche [7]. As in vitro culture environment offers fertile sustenance for a wide range of microorganisms, an unavoidable step, successful culture initiation requires the eradication of all the contamination including filamentous fungi, yeasts, bacteria, viruses, viroids, and micro−arthropods (mites and thrips) which can alter the subsequent growth and development of the explant [8, 9]. Biological contamination is a serious problem in plant tissue culture [10–12]. Contaminants can release metabolites and proteins that affect the plant tissues and alter the composition and/or pH of the culture medium [13]. With a faster growth rate and in conjunction with the nutrient’s availability, contamination colonizes the medium and the explant eventually dies in a matter of days. This obstacle exacerbates financial losses in the commercial sector as well as limits experiment progress in research laboratories. The severity of this issue was corroborated by the adoption of microbiological quality assurance systems (e.g. Hazard Analysis Critical Control Point; HACCP procedures) to succor the requirements of commercial plant tissue culture laboratories [8]. Disinfection efficiency depends on the contamination type (epiphytic or endophytic and expressed or latent), the explant (type, age, size, choice of explant, sampling time, physiological state of the donor plant, and culture condition) as well on the disinfection procedure [14]. Accordingly, various compounds were applied to establish axenic culture e.g. hydrogen peroxide (H_2_O_2_), mercuric chloride (HgCl_2_), silver nitrate (AgNO_3_), silver nanoparticles, calcium hypochlorite (Ca(OCl)_2_), antibiotics, etc. Though, sodium hypochlorite (NaOCl) represents the more considered option for chemical disinfection with a broad antimicrobial spectrum, rapid bactericidal action, solubility in water, and relative stability [15]. There is no standard decontamination protocol. Occasionally, a research laboratory should adjust its own procedure that is well adapted to studied biological material namely, should not affect the viability and the regeneration capacity. Explant health is the main feature determining regeneration potential. Seed germination, seedling growth, and shoot regeneration were negatively affected by the increasing concentration and temperature of NaOCl solution in flax (*Linum usitatissimum*) [16]. A disinfecting agent can alter seed metabolism, trace amounts of NaOCl that remain on the surface of *Lycopersicon esculentum* Mill. seeds after sterilization interfere with subsequent uptake and incorporation of leucine into protein [17]. As well, it can influence the number and size of stomata and cells and the total chlorophyll content [18]. Even further, the disinfection procedure can affect the future development pathway of the explant [19]. Still, the conventional one factor at a time (OFAT) “optimization” way, i.e. by assessing some defined levels of the involved factors, e.g., sterilization agent, concentration, immersion time, temperature, shaking, etc., may give rise to limited results since, presumably, even untested miniature variation could be influential in the corresponding response.

The implication of artificial intelligence−optimization algorithms, a highly potent technology combination, in plant tissue culture has recently emerged with limited application due to the complex definition terms and computational algorithms. Notwithstanding, it was considered to achieve different purposes such as modeling the effects of light and sucrose, optimization of medium culture formulation, prediction and optimization of cell growth, shoot organogenesis, in vitro rooting, and somatic embryogenesis [20–23]. The adoption of modeling and 3D printing technologies for the design and development of a functional plant tissue culture vessel revealed a successful application to prototype novel culture vessels with independently controlled variable fluence rate/spectra LED lighting [24]. Light (quantity and particularly quality) is the main factor that affects photomorphogenesis and with high effect on protocol reproducibility among laboratories [25, 26]. Dissimilar to the classical linear regression−based analysis, the robustness of machine learning methods is that it makes it possible to take into account the overall interaction effect of the different involved variables in a particular event. This is primordial since the plant tissue culture approach encompasses dynamic, non − linear, and non − deterministic processes, therefore, able to interpret highly complex relationships between dependent and independent variables. Artificial neural networks (ANNs) are the most applied for modeling and optimization in plant tissue culture such as radial basis function (RBF), generalized regression neural network (GRNN), probabilistic neural network (PNN), neurofuzzy logic, support vector machine (SVM) with multilayer perceptron (MLP) is the most popular ANN [27]. The superiority of ANNs tool, compared with others modeling technologies such as response surface methodology (RSM), was extensively proved by various reports [28, 29]. ANNs were usually linked to various types of algorithms for optimization purposes. Regarding multi–objectives problems, several multi–objective evolutionary algorithms were developed including multi–objective genetic algorithm (MOGA) [30], nondominated sorting genetic algorithm (NSGA) [31], and fast nondominated sorting genetic algorithm (NSGA–II) [32]. Multi−objective genetic algorithm was suggested as an effective way to aggregate all objectives simultaneously and proposes a reasonable solution to a multi–objective problem following the investigation of a set of solutions, each of which satisfies the objectives at an acceptable level without being dominated by any other solution.

Pistachio (*Pistacia vera* L.), a luxury and high economically important crop from Anacardiaceae family, is cultured mainly in hot and dry climates including Western Asia, Asia Minor, Northern Africa, Southern Europe, and California [33]. A treasure species distinguished by great adaptability to marginal climatic and edaphic conditions such as drought, cold, calcareous, and rocky soils [34]. Though, its culture has been largely restricted, partially, by inefficient methods of vegetative propagation. Various attempts have been reported regarding the adoption of in vitro culture technology that revealed, most often, various physiological disorders (browning, shoot apical necrosis, phenolic compounds exudation, vitrification …) [35, 36]. To those, the hurdle of the culture aseptic initiation was frequently reported which is due to the surface colonizers as well as endogenous contamination [37–39]. Consequently, this species is still underexploited in arid and semi–arid regions.

Some works reported successful decontamination in *Pistacia vera* explants [38, 39]. Nonetheless, the protocol should be adjusted to take precautions against phytotoxicity and ensure high explants’ health which could be accomplished using computer−based tools. Thus, the modeling of surface disinfection protocol of field−derived explants was considered by comparing the effect of three sterilizing agents, their concentration, and the time exposure. Afterward, an optimized combination was defined using multi−objective genetic algorithm aimed to define the lowest effective chemical concentration for the shortest application time.

## Materials and methods

### Plant material, treatments, and in vitro culture conditions

Mature nuts of pistachio (*Pistacia vera* L.) elite Tunisian variety ‘Mateur’ were collected from 35 − year−old trees cultured in Medenine, Southeast of Tunisia. After removing the hull and the shell, the kernels were surface−sterilized under a laminar flow hood (ZHJH−C1214C Vertical Airflow; HEPA Filter) sterilized using UV radiation for 15 min and 75% ethanol (C_2_H_5_OH) immediately before the manipulation. Sterile gloves, continuously sprayed with 75% ethanol, were used. Three sterilizing agents were evaluated, sodium hypochlorite (NaOCl; 3.61% active chlorine) at 0.54, 0.90, and 1.26% (w/v), hydrogen peroxide (H_2_O_2_, 35%) at 5.25, 8.75, and 12.25%, both for 10, 20, and 30 min, and mercury (II) chloride (HgCl_2_; HiMedia, India) at 0.05, 0.1 and 0.2% for 5, 10 and 15 min (Table 1). The chemicals were added to the sterile distilled water (SDW) after cooling to room temperature under the laminar flow hood. Heating NaOCl solutions may cause unpredictable changes to the concentration of available Cl^−^ [40]. The ranges of parameters were designated based on our preliminary experiments as well as considering the literature. Glass jars (300 ml) were used with 150 ml of disinfection solution. All the materials, including the glass jars, forceps, scalpel handle, and the filter paper (to eliminate the excess SDW from explants), were sterilized using an autoclave at 121 °C for 20 min. Disposable sterile surgical blades were used. Soaking of forceps and scalpel in 100% ethanol and flame sterilization are continuously carried out during the manipulation. Likewise, sterile filter paper layers were changed regularly between treatments. Stainless steel infuser was used to facilitate the recuperation of explants. New sterilizing and SDW solutions were reserved for each treatment to avoid any concentration of the chemical product. Explants were washed four times in SDW and inoculated in full strength Murashige and Skoog (MS) medium [41] devoid of plant growth regulators, supplemented with 3% (w/v) sucrose (HiMedia, India), 0.75% (w/v) agar (Biolife, Italy), 100 mg L^− 1^ ascorbic acid and distributed in glass tubes (200 mm × 25 mm) hermetically sealed using parafilm. The two cotyledons of each kernel were incubated separately (with one of them including the embryonic axis). pH of the medium was adjusted to 5.8 ± 0.1 prior to autoclaving at 121 °C for 20 min. A total of 522 cultures, including the control and the validation assay, were devoted to the current study with eighteen explants for each treatment and one explant/tube to avoid the risk of spreading contamination. Explants treated with SDW for 20 min served as the control. Cultures were incubated in a growth incubator at a controlled temperature of 25 ± 1 °C with a 16 h photoperiod (40 μmol m^− 2^ s^− 1^) for 20 days. Routinely monitoring of contamination was carried out during incubation, usually, detectable by the ‘halo’ effect around the contaminated explant, turbidity of the medium, cell destruction, etc. Ultimately, disinfection efficiency and potential negative disinfection impact were recorded by visual examination. The negative disinfection effect was evaluated considering only the contamination−free cultures.

**Table 1:** Decontamination procedures of *P. vera* explants with the corresponding disinfection efficiency and negative disinfection effect for experimental and predicted values

		Independent variables ^a)^				Dependent variables					
Run	Code	x_1_	x_2_	x_3_	x_4_	Disinfection efficiency (%)			Negative disinfection effect (%)		
						Actual	predicted	Residual	Actual	predicted	Residual
1	T0 (C)	0	0	0	20	0.00	2.62	−2.62	–	–	–
2	T1	0.54	0	0	10	44.44	73.69	−29.25	0.00	−17.96	17.96
3	T2	0.54	0	0	20	27.78	26.80	0.97	0.00	6.94	−6.94
4	T3	0.54	0	0	30	55.56	29.24	26.32	0.00	20.12	−20.12
5	T4	0.90	0	0	10	72.22	68.95	3.27	0.00	−11.25	11.25
6	T5	0.90	0	0	20	72.22	59.76	12.46	7.69	−11.52	19.21
7	T6	0.90	0	0	30	50.00	44.14	5.86	0.00	8.05	−8.05
8	T7	1.26	0	0	10	44.44	46.57	−2.12	0.00	5.19	−5.19
9	T8	1.26	0	0	20	72.22	72.78	−0.55	0.00	5.92	−5.92
10	T9	1.26	0	0	30	77.78	77.27	0.51	14.29	9.96	4.33
11	T10	0	5.25	0	10	86.67	88.09	−1.43	0.00	−2.67	2.67
12	T11	0	5.25	0	20	83.33	87.28	−3.94	6.67	19.88	−13.22
13	T12	0	5.25	0	30	83.33	79.80	3.54	46.67	42.93	3.74
14	T13	0	8.75	0	10	77.78	74.38	3.40	28.57	18.49	10.08
15	T14	0	8.75	0	20	95.24	93.13	2.10	20.00	23.28	−3.28
16	T15	0	8.75	0	30	72.22	73.24	−1.02	46.15	45.34	0.82
17	T16	0	12.25	0	10	66.67	68.70	−2.03	8.33	15.01	−6.68
18	T17	0	12.25	0	20	100.00	94.02	5.98	11.11	12.39	−1.28
19	T18	0	12.25	0	30	89.47	88.80	0.67	23.53	18.68	4.85
20	T19	0	0	0.05	5	100.00	105.35	−5.35	38.89	32.30	6.59
21	T20	0	0	0.05	10	100.00	98.48	1.52	44.44	42.27	2.18
22	T21	0	0	0.05	15	100.00	94.90	5.10	35.29	52.14	−16.84
23	T22	0	0	0.10	5	100.00	96.30	3.70	22.22	48.45	−26.23
24	T23	0	0	0.10	10	100.00	92.63	1.48	100.00	60.53	39.47
25	T24	0	0	0.10	15	100.00	104.76	−4.76	52.94	64.75	−11.81
26	T25	0	0	0.20	5	100.00	83.73	16.27	83.33	78.71	4.63
27	T26	0	0	0.20	10	100.00	105.48	−5.48	100.00	80.24	19.76
28	T27	0	0	0.20	15	100.00	134.70	−34.70	88.24	76.73	11.51

^a)^ x_1_: NaOCl (% w/v), x_2_: H_2_O_2_ (%), x_3_: HgCl_2_ (%), x_4_: Immersion time (min), C: control

### Artificial neural network model

The ANN was developed based on four inputs, three sterilizing agents at different concentrations and the immersion time, and two outputs, disinfection efficiency (DE) and negative disinfection effect (NDE). A multilayer perceptron (MLP) model was implemented with hyperbolic tangent sigmoid transfer function in the hidden layer and linear transfer function in the output layer. The network was trained with Levenberg–Marquartd back−propagation algorithm. Twenty–eight treatments, including the control, were applied (Table 1), divided randomly into 3 datasets, with 70% (20 samples) for training, 15% (4 samples) for validation, and 15% (4 samples) for testing dataset. All the data were normalized between − 1 and 1 using Eq. (1) to attain dimensional consistency of the parameters and to ensure compatibility with the adopted transfer function [42]:

1 $$Mi=\frac{\left({M}_{max}-{M}_{min}\right)\left({N}_i-{N}_{min}\right)}{N_{max}-{N}_{min}}+{M}_{min}$$

Where *M*_*i*_ is the normalized value, *M*_*max*_ and *M*_*min*_ are the maximum and the minimum values of the scaling range, *N*_*i*_ is the actual data to be normalized, *N*_*max*_ and *N*_*min*_ are the maximum and minimum values of the actual data.

Afterward, the developed model was transformed into a mathematical equation, through the weights and biases in conjunction with the transfer functions:

2 $$\mathrm{DE}\ \left(\%\right)/\mathrm{NDE}\ \left(\%\right)= purelin\left(\left[{\sum}_{i=1}^N{w}_i^2 tansig\left({\sum}_{j=1}^J{w}_{i,j}^1{x}_j+{b}_{1i}\right)\right]+{b}_2\right)$$

$$\mathrm{With}\ tansig(x)=\frac{2}{1+{e}^{\left(-2x\right)}}-1$$

$$purelin(x)=x$$

Where; *x* is the input variables, *N* is the number of neurons, *J* is the number of input variables, *w*^*1*^ is weight of hidden layer, *w*^*2*^ is weight of the output layer, *b*_*1*_ is bias of the hidden layer, and *b*_*2*_ is bias of the output layer.

The prediction performance of the developed model was statistically evaluated in terms of the root mean squared error (*RMSE*) [43] and the coefficient of determination (*R*^*2*^) [44] values as follows:

3 $$RMSE=\sqrt{\frac{1}{n}{\sum}_{i=1}^n{\left({x}_i-{x}_{ik}\right)}^2}$$

4 $${R}^2=1-\frac{\sum_{i=1}^n\ {\left({x}_i-{x}_{ik}\right)}^2}{\sum_{i=1}^n\ {\left({x}_{ik}-{x}_z\right)}^2}$$

Where, *x*_*i*_ is predicted value; *x*_*ik*_ is the experimental or actual value; *x*_*z*_ is the mean of experimental value, and *n* is the number of observations.

### Optimization of disinfection process

To achieve the main goal of the current work, multi−objective genetic algorithm (MOGA) solver was applied to balance the two considered aspects simultaneously i.e. maximize the disinfection efficiency and minimize the negative disinfection effect. As there are two outputs, two different objectives functions were defined by the developed ANN, both fit Eq. (2), and represent an appropriate approximation of the functional relationship between the inputs and the outputs variables. These two equations were introduced as a fitness function for the optimization step. The population type was a double vector with the creation, selection, mutation, and crossover functions were feasible population, tournament, adaptive feasible, and intermediate, respectively, completed with the following parameters: population size: 50, crossover fraction: 0.8, migration fraction: 0.2, stopping criteria: generations: 400, and stall generations: 100.

### Statistical analysis

Matlab®8.3 (R2014a, The Mathworks Inc., Natick, USA) was used for the modeling and the optimization. Data were subjected to multivariate ANOVA analysis and the means were separated by Duncan test (*P* < 0.05) using IBM SPSS Statistics v.25.0 for Windows.

## Results and discussion

### Disinfection process

Contamination hazard is among the more destructive limitations in plant tissue culture, commonly encountered in worldwide involved laboratories, regardless of its source (biological material, inappropriate manipulation, …). A solid understanding of both the type and the potential sources of contaminants is required to prevent culture failure. Rigorous instructions should be respected in this technology. Otherwise, the culture will be discarded. Handled by an experienced manipulator in a specialized laboratory, contamination could be effectively managed to be overcome.

In the matter of surface disinfection, a total of twenty−seven treatments were evaluated for pistachio seeds disinfection in the current experiment. The studies concerned seed–derived material are with high–priority as the genetic variation, the main characteristic of the fertilization process, may generate a biological material with new interesting characters. Equally, *P. vera* is commonly used as a rootstock in this species during field or micrografting [45]. Furthermore, any advancement recorded in seed studies could be, high presumed, extended to tree–derived explant.

**Fig. 1:**
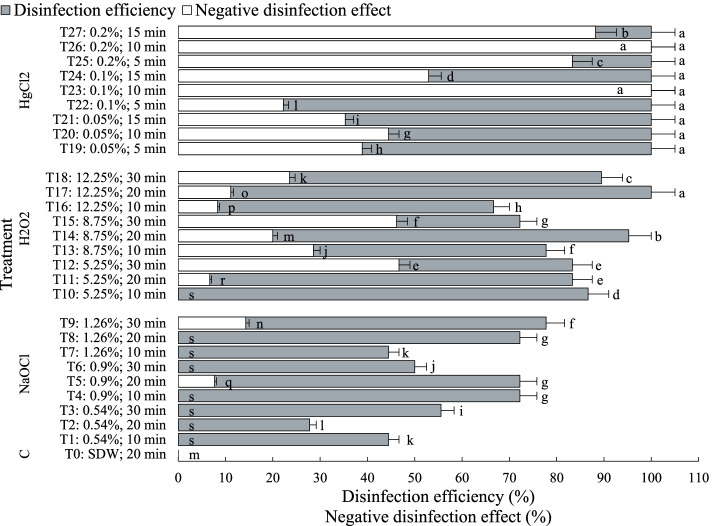
Disinfection efficiency and negative disinfection impact of *P. vera* explants using three chemicals at different concentrations and immersion time, NaOCl (0.54, 0.9, 1.26% w/v; 10, 20, 30 min), H_2_O_2_ (5.25, 8.75, 12.25%; 10, 20, 30 min), and HgCl_2_ (0.05, 0.1, 0.2%; 5, 10, 15 min) after 20 days of culture. Different letters indicate a significant difference using Duncan’s test at *P* < 0.05

Multivariate ANOVA analysis reflected a high significant (*p* < 0.001) effect for the three implicated parameters viz. sterilizing agent, its concentration, and the processing time, as well as all their interactions (Table 2). A 100% contamination was registered in the control culture (treated with SDW). In respect of disinfection efficiency, HgCl_2_ appears the most effective (100%), then H_2_O_2_ (66–100%), followed by NaOCl (27–77%) (Table 1, Fig. 1). Synchronously, the direct contact with disinfectants affected negatively the explants’ health at different levels during some treatments. The undesirable effects (NDE) included chlorosis (or yellowing), hard embryo germination, embryo deformation, and browning tissue. The same order of chemical agent aforementioned was recorded i.e. a low NDE with NaOCl (0–14%), a moderate effect with H_2_O_2_ (6–46%), and pronounced damage with HgCl_2_ (22–100%). Thus, the best result, for both evaluated aspects, was obtained with H_2_O_2_ at 5.25 and 12.25% for 10 min (T10) and 20 min (T17), respectively. No NDE was obtained with T10, giving 86.6% of healthy sterile explants. Equally, a very close value was recorded with T17 (88.9%) following the ignoring of a small fraction of NDE. With stem segments, explants of *P. vera* were subjected to (*i*) 0.1% HgCl_2_ and 0.3% CaCl_2_ both for 10 min and (*ii*) 2.6% (w/v) NaOCl for 10 min with a few drops of a wetting agent. Both of them gave about 96% contamination−free culture [39]. Increasing NaOCl concentration from 5 to 20% (v/v) for 20 min, in apical tips treatment from adult male pistachio, showed better decontamination, meantime, the proportion of survived explants was negatively affected [38]. In *Melia azedarach* L. culture, the lowest contamination and browning response with the highest percentage of callus induction and growth were obtained with benomyl (a systemic fungicide) pretreatment (2 g L^− 1^) for 2 h and 7% H_2_O_2_ for 10 min and 2% (w/v) NaOCl for 12 min [46]. Hence, generally, variable but convergent levels of chemicals are recommended for effective decontamination.

**Table 2:** Multivariate ANOVA of between–subject effects for disinfection efficiency (DE) and negative disinfection effect (NDE) of *P. vera* explants with different decontamination treatments

Source	Dependent variable	Type III Sum of Squares	Mean Square	F	Sig.
Corrected Model	DE	52,717.660^a^	1952.506	3215.882	.000
	NDE	95,350.715^b^	3531.508	3.097E+ 29	.000
Intercept	DE	309,231.954	309,231.954	509,321.611	.000
	NDE	93,306.460	93,306.460	8.183E+ 30	.000
Sterilizing agent	DE	15,848.327	7924.163	13,051.522	.000
	NDE	38,811.509	19,405.755	1.702E+ 30	.000
Concentration	DE	920.976	460.488	758.448	.000
	NDE	4194.618	2097.309	1.839E+ 29	.000
Immersion time	DE	896.344	224.086	369.082	.000
	NDE	7900.709	1975.177	1.732E+ 29	.000
Concentration * Immersion time	DE	4805.943	600.743	989.456	.000
	NDE	4906.072	613.259	5.378E+ 28	.000
Sterilizing agent * Immersion time	DE	564.100	282.050	464.552	.000
	NDE	1578.866	789.433	6.923E+ 28	.000
Sterilizing agent * Concentration	DE	2281.543	570.386	939.456	.000
	NDE	3822.137	955.534	8.380E+ 28	.000
Sterilizing agent * Concentration * Immersion time	DE	506.855	126.714	208.704	.000
	NDE	1197.868	299.467	2.626E+ 28	.000
Error	DE	34.000	.607		
	NDE	6.386E–25	1.140E–26		
Total	DE	555,182.632			
	NDE	178,015.050			
Corrected Total	DE	52,751.660			
	NDE	95,350.715			

^a^R − squared = .999 (Adjusted R − squared = .999)

^b^R − squared = 1.000 (Adjusted R − squared = 1.000)

**Fig. 2:**
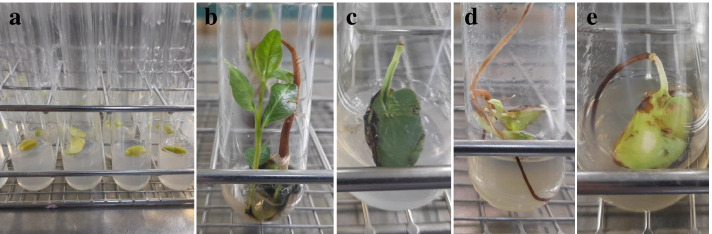
Effect of some disinfection procedures on *P. vera* explants after 20 days of culture: **a** Explants at day 0 of incubation; **b** germination of healthy zygotic embryos (T3:0.54% NaOCl, 30 min); **c** cotyledon explant with dark green color (T5: 0.9% NaOCl, 20 min); **d** deformed embryo germination (T27: 0.2% HgCl_2_, 15 min); **e** explant with chlorosis and browning symptoms (T26: 0.2% HgCl_2_, 10 min)

Theoretically, regardless of the potential induced damage, a more concentrated disinfectant, escorted or not with increased exposure time, will ensure more microorganisms eradication. This is well noticed, for instance, during in vitro sterilization of chrysanthemum including different concentrations of six sterilizing agents and the immersion time [47]. Yet, this notion was refuted here as more accentuated treatment does not necessarily show better asepsis. As Fig. 1 displays, there are fluctuations in the disinfection response even with the same chemical agent concentration noticed mainly with NaOCl and H_2_O_2_. Some effects of appraised disinfection procedures are presented in Fig. 2.

During disinfection processes, the biological activity of cells should not be affected and only contaminants should be eliminated. Although it seems a simple step, decontamination in some species is extremely difficult. In many cases, anti−microbial treatments only inhibit contaminants and low levels of these latter persist. Detection of latent contamination, namely, without visible symptoms on explant and/or visible growth on medium culture, may involve screening using general and semi−selective microbial growth media or serological and PCR − based molecular techniques for specific pathogens. Nonetheless, it is often difficult to detect low numbers of latent bacterial contaminants [8]. The latent aspect could be correlated to the absence of some growth factors/vitamins in the plant culture medium required for bacterial growth [48]. Moreover, the acidity of the medium and the release of antibacterial exudates by the plant cells/tissues can also suppress bacterial growth [49]. This kind of contamination could proliferate following the transfer from one stage to another which involved usually adjustment in the nutrient regimes.

Disinfection treatment can highly affect the subsequent response of the tissue, alongside a multitude of factors including even the explant orientation (vertical, inverted vertical, horizontal, and abaxial or adaxial face), which substantiates its importance. The use of hot water and plant preservative mixture (PPM), a broad–spectrum biocide/fungicide for plant tissue culture, improves the rate of bud germination and differentiation from 4 to 50% in ginger (*Zingiber officinale***)** explants [19]. By testing two sterilization procedures for stem tips culture in *P. vera*, it was described that NaOCl was more conducive to rosette development (75%) but did not significantly differ from HgCl_2_ (66.7%) [39].

As above–stated, a wide range of surface disinfectants with varying degrees of effectiveness have been used in plant tissue culture, often with a few drops of a wetting agent (e.g. Tween 20). Still, NaOCl is the most widely applied compound [50, 51]. Based on its concentration, the germicidal effect of NaOCl was attributed predominantly to hypochlorous acid (HOCl) in diluted solution [52] and to its high pH (12.5–13.5) and hypochlorite ion (ClO^−^) oxidizing agent in concentrated form [53]. ClO^−^ has a poor germicidal activity due to its inability to diffuse through the microbial plasma membrane and it exerts an oxidizing action only from outside of the cell. HOCl can penetrate across the cell wall and the lipid bilayer in the plasma membrane by passive diffusion due to its electrical neutrality, thus, can attack the microbial cell both from the outside and inside the cell, which is responsible for the potent germicidal activity of this agent. Both substances give rise to the inhibition of enzyme activity essential for the growth, damage to the membrane and DNA, and possibly an injury to membrane transport [15]. The pH adjusting (to pH = 7 and 10) reduced significantly the microbial contamination in *Melia azedarach* L. culture, but, adversely influenced the explant viability and callus induction and growth [46]. A positive effect was accorded to this disinfection agent during micropropagation of *Kalanchoe tubiflora* (Harvey) Hamet which has been assumed to correlate with the positive influence of stress (associated with explants disinfection) [54]. Chemical sterilization, using NaOCl, was proved to be useful as a replacement for thermal sterilization of nutrition media [55]. Chlorine dioxide was also used to sterilize the medium for tissue culture of potato (*Solanum tuberosum L*.) [56]. NaOCl can alleviate significantly the inhibitive effect of salt stress during the germination phase. Added to the germination solution, *Allium cepa* L. seed treated with a 0.1% NaOCl + 0.225 M NaCl for 7 d showed an improvement in the germination percentage, the radical length and number, and the fresh weight compared with the salt treatment [57]. In another aspect, NaOCl and H_2_O_2_ were described as effective for releasing dormancy of imbibed wild oat (*Avena fatua* L.) seeds via a modification of the properties of the hull and seed coat membranes and in the provision of additional oxygen to the seeds [58]. This fact was evidenced in this experiment. Pistachio zygotic embryos were able to respond to external stimulants and germination was noticed in all explants considering that the cultures were induced with dormant seeds. Besides its effect as a disinfectant agent, it was reported that NaOCl can be used as a dormancy−breaking agent by decomposing germination inhibitors [59], scarification of the seed coat [16], and increasing α–amylase activity [60].

Mercuric chloride is a highly toxic chemical reagent for humans and plant tissues [61–63]. Heavy metals, such as mercury, are known for their immunotoxic and neurotoxic properties and are environmental pollutants [64]. Nevertheless, this compound was used in various works for disinfection purposes in in vitro plant culture. Its toxic potential has been confirmed, in the present experiment, especially with the application of a high concentration for a long immersion time (Figs. 1 and 2). Whereas, H_2_O_2_ is a non–phytotoxic compound due to the activity of plant peroxidases and catalases allowing its transformation into water and oxygen [65]. In agreement, a relatively moderate negative impact was noticed here with this chemical.

As aforementioned, several disinfection protocols were proposed. The one factor at a time (OFAT) approach was commonly considered for the “optimization” of the process. For instance, for the decontamination of shoots or apical tips from male *Pistacia vera* L. cv. ‘Atlı’, NaOCl was applied at four concentrations 5, 10, 15, and 20% (v/v) for 10 min as the first step. Then, 10% NaOCl was selected for combination with five immersion times 5, 10, 20, 30, and 40 min. Subsequently, the presterilized explants (10% NaOCl for 30 min) were subjected to 10% H_2_O_2_ for 5 and 10 min [38, 66]. This approach is conventionally adopted, although, it does not allow highlighting the interactive effect between the different factors.

### Artificial neural network modeling

**Fig. 3:**
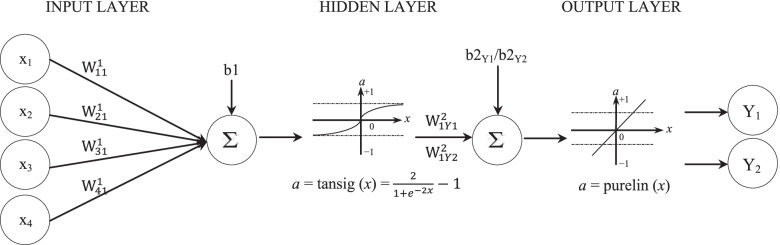
Details of developed feed–forward back–propagation neural network (Topology: 4–10 − 2): input layer with 4 neurons, one hidden layer with 10 neurons (only neuron N°1 was presented), and output layer with 2 neurons. W^1^: weight of hidden layer, W^2^: weight of output layer, b1: bias of hidden layer, b2: bias of output layer, x: inputs (x_1_: NaOCl (%), x_2_: H_2_O_2_ (%), x_3_: HgCl_2_ (%), x_4_: immersion time (min)), Y: outputs (Y_1_: disinfection efficiency (%), Y_2_: negative disinfection effect (%))

To simulate the connection between the three sterilizing chemicals, their concentrations, and the immersion time, on one side, and the disinfection efficiency and the negative disinfection effect, on the other side, a multilayer perceptron (MLP) model was developed. The ANN consisted of three layers, the input layer with 4 neurons, the hidden layer with 10 neurons, and the output layer with 2 neurons (Fig. 3). The number of neurons in the hidden layer was decided using the trial−and − error method by changing the number of neurons until a maximum regression *R* and minimum mean squared error (MSE) were obtained. Therefore, the ANN topology was 4–10 − 2. Determining the construction of the MLP has the main function in its performance [67]. Each neuron in the hidden and the output layer has a bias (*b*) value and is linked to the previous layer’s neurons with an interconnection having a certain weight (*w*) and, altogether, the generated structure creates the network. The sizes for the weights matrices are 10 × 4 and 10 × 1 for both evaluated parameters, for joining the input layer to hidden layer and the hidden layer to output layer, respectively, while the size of the biases matrices are 10 × 1 and 1 for the neurons of the hidden layer and the output layer, respectively. The ANN dimensions were given in Eqs. (5, 6, 7, 8).

5 $$IW1\left[\begin{array}{cccc}-2.2264843404709231\ & 1.4445054452140973\ & 0.37347240570903362& 1.7912129167797899\\ {}0.28373790392803888\ & -2.4322869476045663& -0.49844554568205773& 1.285538179348072\\ {}-1.577021437225925\ & -2.3216990424583965& -1.0439509360936723& -0.49127974227126764\\ {}-1.3929110122378521\kern0.5em & -1.5862753950671646& -0.6943257810737461& 0.92230670254520253\\ {}\ 0.0062537023069687697\ & 1.1089013107640835\ & 0.70865214563352252& 1.1472529657345023\\ {}-1.992372972284326\ & -0.82189187786987539& 1.527011149326817& -0.18686885970967243\\ {}-1.1378541924874293\ & 0.84648602435997011& -0.9780357313064737& -1.3630683902071483\\ {}\ 1.5020518212917611\ & 0.32509362788792218& -1.5693931358736695& -0.031089357432888279\\ {}-1.4882883913438978\ & -2.9651377089231907\ & 2.199846446705342& -0.43752062219922738\\ {}1.3599011247022701\ & -0.81015345267550232& 2.9754824151678636& -1.9571431435625306\end{array}\right]$$

6 $$b1\left[\begin{array}{c}2.7373703731067001\\ {}-0.83325876887422079\\ {}2.171582970034827\\ {}0.56212948576185007\\ {}1.0424070358882143\\ {}-0.54324401496903252\\ {}-1.1022234392936949\\ {}2.0378619763856407\\ {}1.2672225714710146\\ {}2.9832316738791187\end{array}\right]$$

7 $$LW2\ (Y1)\left[\begin{array}{c}\ \\ {}-0.35262980357087659\ \\ {}-0.24318178776467936\\ {}-0.79248319436380665\\ {}0.82630798614474876\\ {}1.796820588074038\\ {}0.21420000089354962\\ {}0.60020942311500314\\ {}0.40809858306703195\\ {}0.57830471663552308\\ {}1.2020209830201682\end{array}\right];(Y2)\left[\begin{array}{c}-0.12048113211448197\ \\ {}0.34901295143434985\\ {}0.63326846110801793\\ {}0.042982012720122198\\ {}-0.68987705311744119\\ {}0.94749566689285258\\ {}-0.43511551317049457\\ {}-0.56189528509021669\\ {}-0.83086076761510286\ \\ {}-0.73536668215158751\end{array}\right]$$

8 $$b2\ (Y1)\left[0.35872919821907595\right];(Y2)\ \left[-0.19926566214623609\right]$$

**Fig. 4:**
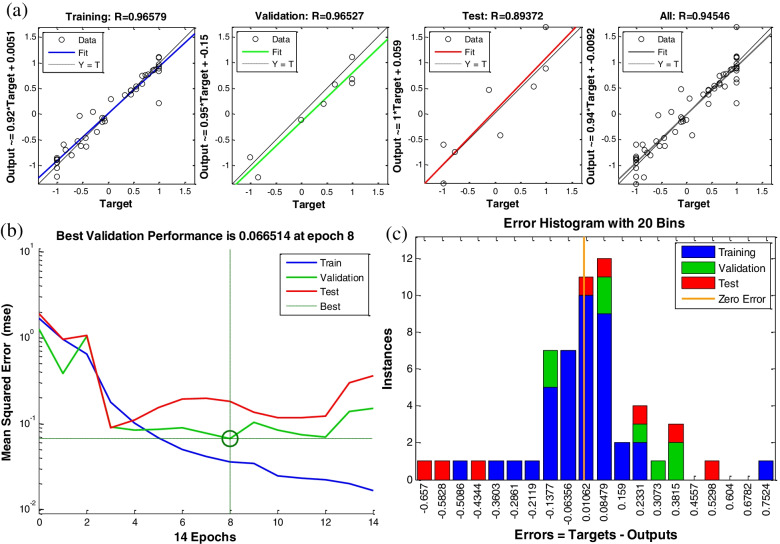
**a** Regression analysis for training, validation, testing, and all data sets, with an appropriate matching between the target and the output values, **b** performance, and **c** error histogram of the implemented ANN model

Post−training analysis showed *R* values: 0.9658, 0.9653, 0.8937, and 0.9454 for training, validation, testing, and all, respectively, revealed a good correlation between the predicted (output) and the actual (target) data (Fig. 4a). The performance evaluation, in terms of MSE, showed that the network manifests improvement and the best validation performance was obtained at 8 epochs with an *MSE* value of 6.65E− 02 (Fig. 4b). Below 8 epochs, *MSE* is high. Whereas, with a higher epoch number, the *MSE* of training data decreased, reflecting a network overfitting. By considering *R* for the training dataset (0.9658), thus, the best performance is automatically selected and the model is assumed to be adequate for data prediction. The predictive capacity of the model can be assessed also considering calculated parameters with 11.09 and 13.5 for *RMSE* and 0.804 and 0.839 for *R*^*2*^ values for DE and NDE, respectively (Table 3). Figure 4c shows that the data fitting errors, for training, validation, and testing are distributed within a reasonably good range and are very close to zero.

**Table 3:** Calculated predictive capacity of ANN model

Calculated predictive capacity	DE	NDE
*RMSE*	11.088993	13.5210135
*R*^*2*^	0.80406535	0.83894514

*RMSE* Root mean squared error; *R*^*2*^ Coefficient of determination

### Optimization using multi–objective genetic algorithm

Dissimilar to single–objective problems, in multi–objective situations, it is not easy to find the optimal solution since the objective functions are usually in conflict with each other, that is means improving one will negatively affect the other. Thus, a balanced solution must be established. The presented conflict in the current experiment is to define the optimal combination of inputs to ensure the maximum value for the objective function of decontamination percentage output and, simultaneously, the minimum value for the objective function of negative disinfection effects. Following multi−objective genetic algorithm running, the proposed combination included the three sterilizing agents at different levels: 0.563%, 12.232%, and 0.068% for NaOCl, H_2_O_2_, and HgCl_2_, respectively, with an immersion time of 5.022 min. Subsequently, the validation assay has proved the accuracy of this tool with a high coincidence between the expected and the obtained values with 100% contamination−free culture and 7.1% only of negative disinfection effects. In the same framework, optimizing of in vitro sterilization of chrysanthemum was proposed using multilayer perceptron non–dominated sorting genetic algorithm–II (MLP–NSGAII) encompasses seven inputs, viz. six sterilizing agents and the immersion time, and two outputs including contamination frequency and explants viability [47]. They suggested MLP–NSGAII as an efficient method in different areas of in vitro culture.

## Conclusion

Machine learning models and optimization algorithms, revolutionary computational technologies, have opened wide perspectives in the field of plant tissue culture, especially for recalcitrant species. This work represents a case study concerning the effectiveness of these tools and the successful implementation of multi–objective genetic algorithm that can be extended to any nonlinear multivariate processes. An optimized decontamination treatment consisting of 0.56% NaOCl, 12.23% H_2_O_2_, and 0.068% HgCl_2_ for 5.022 min was designed for in vitro *P. vera* seeds culture. The established protocol will be assessed for other types of explants in subsequent manipulations. It could be either efficient or needs some adjustment considering that the adjoining features can diverge among explants. Meanwhile, underpinned by suitable laboratory practices, the present investigation spotlighted the ingenuity of artificial intelligence technology to manage such steps regardless of the concerned explant. Similarly, optimization algorithms will have an unprecedented impact in further intricate tasks, for instance, in plant growth regulators management, the main decisive molecules for in vitro cell fate acquisition, an extremely precise aspect in plant tissue culture.

## Data Availability

All data concerning the current work were included in the manuscript. Other materials and any clarification that support the findings of this study are available from the corresponding author on reasonable request.
